# [^18^F]F-FAPI-74 PET/CT for gonadal and extragonadal germ cell Tumors: a pilot study

**DOI:** 10.1186/s13550-025-01347-y

**Published:** 2025-12-20

**Authors:** Marc Kidess, Sophie C. Siegmund, Lena M. Unterrainer, Philipp Kazmierczak, Marcus Hentrich, Lennert Eismann, Yannic Volz, Benedikt Ebner, Julian Hermans, Nikolaos Pyrgidis, Maria Apfelbeck, Gabriel T. Sheikh, Rudolf A. Werner, Frederick Klauschen, Christian G. Stief, Stephan T. Ledderose, Michael Chaloupka

**Affiliations:** 1 Department of Urology, LMU University Hospital of Munich, Marchioninistr. 15, Munich, 81377 Germany; 2 Department of Nuclear Medicine, LMU University Hospital of Munich, Munich, Germany; 3 Department of Radiology, LMU University Hospital of Munich, Munich, Germany; 4 Department of Medicine III, Red Cross Hospital Munich, Munich, Germany; 5 Insitute of Pathology, LMU University Hospital of Munich, Munich, Germany; 6 Bavarian Cancer Research Center (BZKF), partner site, Munich, Germany; 7 The Russel H. Morgan Department of Radiology and Radiological Sciences, Division of Nuclear Medicine, Johns Hopkins School of Medicine, Baltimore, USA

**Keywords:** Testicular cancer, FAPI, Staging, Imaging, PET

## Background

Testicular Cancer (TC) is the most common cancer in young men with a 5-year survival rate of 95% [1]. This is most likely due to the availability of successful therapies even in metastatic stages, including surgery, chemotherapy and radiotherapy. The majority of TC are germ cell tumors (GCT), which can be categorized as seminomatous and nonseminomatous GCTs. At the time of diagnosis, 70–75% of patients present with stage I TC (localized disease), 20% with stage II (metastatic disease in the retroperitoneal lymph nodes) and 10% with stage III (distant metastasis) [2]. 5% of GCT patients present with primary extragonadal tumor [3]. While computed tomography (CT) is recommended for (re-)staging of patients with TC, ^18^F-fluorodeoxyglucose-positron emission/computed tomography ([^18^F]FDG PET/CT) is an option in the evaluation of residual tumor masses after chemotherapy in patients with seminomatous GCT [4].

PET imaging has the advantage of combining conventional imaging with molecular imaging to detect occult metastases in other urologic malignancies [5]. [^18^F]F-FAPI-74 PET/CT is a new imaging modality, targeting the fibroblast-activation-protein (FAP), which is expressed by cancer-related fibroblasts in the tumor microenvironment of solid malignancies and was initially described in 1988 [6]. FAP can be targeted by several tracers and the use of FAPI PET/CT has shown promising results for staging in other malignancies like bladder cancer or penile cancer [7, 8]. FAPI PET/CT has not yet gained approval for routine clinical use in any cancer entity. However, existing practice guidelines are designed to help practitioners provide appropriate care for their patients [9].

To date, there is only one case report describing the use of FAPI PET/CT for staging in a patient with GCT [10]. This single-center feasibility study is the first investigating the potentials of [^18^F]F-FAPI-74 PET/CT scan for staging of patients with testicular or extragonadal GCT.

## Materials and methods

### Patient selection and PET/CT scan

GCT patients planned for chemotherapy or residual tumor resection (RTR) after prior chemotherapy received [^18^F]F-FAPI-74 PET/CT prior to re-treatment. Tracer uptake was measured in suspicious tumor masses such as primary tumor site, lymph nodes or residual tumor masses. Imaging results were compared to histopathology. Regions of suspicious lesions were determined on imaging and the lesions with the highest standardized uptake value (SUV_max_) were assessed. None of the participants had distant metastasis.

In accordance with § 13(2b) of the German Pharmaceuticals Act, the production of [^18^F]F-FAPI-74 was carried out under the direct responsibility of the prescribing physician. The precursor FAPI was supplied in GMP quality by SOFIE (Dulles, Virginia, USA).

[^18^F]F-FAPI-74 was synthesized on-site using a 16.5 MeV PETtrace cyclotron (GE Healthcare, Uppsala, Sweden) via the ^18^O(p, n)^18^F nuclear reaction, achieved by proton bombardment of enriched H_2_^18^O (Rotem Industries, Arava, Israel). The radiosynthesis of [^18^F]F-FAPI-74 was conducted on an AllinOne module (Trasis, Liège, Belgium) with a commercially available FAPI-74 cassette and reagent kit (Trasis, Liège, Belgium). The process involved preformulation of aluminum chloride in dimethylsulfoxide and acetonitrile, followed by pH adjustment to 4–5 using ascorbic acid. Fluoride, with starting activities of 47.2 ± 13.4 GBq, was captured on a QMA cartridge and eluted using a NaCl-based solution. The radiolabeling reaction proceeded at 70 °C for 10 min, and the crude product was subsequently purified via solid-phase extraction (SPE) before formulation in a sodium ascorbate-containing matrix. This streamlined process resulted in radioactivity yields (RAY) of 63.0 ± 9.2% (*n* = 36) with a radiochemical purity (RCP) of 99.8 ± 0.1% (*n* = 36), achieved without HPLC purification. The final product was sterile-filtered through a Merck Cathivex-GV filter and dispensed under cleanroom class A conditions. At the end of synthesis (EOS), the specific activity ranged from 1729 GBq/µmol to 10,286 GBq/µmol. Quality control of the final product was conducted in compliance with local regulations.

PET/CT scan was performed 60 min after tracer injection. A mean activity of 228 MBq (range 200–291) was injected intravenously. All patients were injected additionally with furosemide (Furosemid-ratiopharm 20 mg/2 ml injection solution, ratiopharm GmbH, Ulm, Germany) for radiation protection as well as to reduce urinary activity in the renal pelvicalyceal system if no contraindication was given. All patients received a contrast enhanced CT (ceCT) either as part of the [^18^F]F-FAPI-74 PET/CT scan or as a separate additional scan. Findings in conventional CT were correlated with PET/CT scans. Analysis of PET scans were performed using Hermes Hybrid viewer (Hermes Medical Solutions, Stockholm, Sweden). PET-positive lesions were identified by increased tracer uptake above the background.

This analysis was performed in compliance with the principles of the Declaration of Helsinki and its subsequents amendments and prospective registry study of data was approved by the institutional ethics board of the LMU Munich (IRB 24–0255).

### Immunohistochemical analysis and semiquantitative analysis of FAP expression

Immunoreactivity for FAP was assessed using immunohistochemistry on formalin-fixed, paraffin-embedded (FFPE) tissue samples. Antigen retrieval was performed through heat treatment with Target Unmasking Fluid (PanPath B.V., Budel, Netherlands; Z000R0000). The slides were then incubated at room temperature for 60 min with rabbit monoclonal anti-human primary antibodies against FAP (1:120; Abcam, Berlin, Germany; EPR20021). Bound antibodies were detected using the ImmPRESS Anti-Rabbit IgG Polymer Kit (Vector Laboratories, Newark, CA, USA; MP-7401). Staining was visualized with DAB+ (Agilent Technologies; K3468) as the chromogen. Finally, sections were counterstained with hematoxylin (Vector Laboratories; H-3401), dehydrated, and mounted.

FAP staining was assessed as previously described [11]. Briefly, both the staining intensity and the percentage of tumor stroma involved were evaluated in FAP-stained sections using a two-digit scoring system: one digit for intensity and the other for the percentage of stained tumor stroma. The percentage of immunoreactive stromal cells was scored semiquantitatively as 0 (absent or < 1%), 1 (1–5%), 2 (6–33%), 3 (34–66%) and 4 (67–100%). The staining intensity was also graded semiquantitatively on a scale of 0 to 3 (0 = absent staining, 1 = weak staining, 2 = moderate staining, 3 = strong staining).

The final interpretation was determined by combining the intensity score with the tumor stroma percentage score as follows:

- 11–12: negative.
- 13, 14, 21: weak positive.
- 22, 23: moderate positive.
- 24, 31–34: strong positive.

The correlation between FAP expression score and SUV_max_ was calculated using the Pearson correlation coefficient (r). The FAP expression of each patient specimen was correlated with all SUV_max_ detected in the patient’s PET/CT scan.

## Results

Overview about the baseline characteristics, oncological data and results is displayed in Table 1.

**Table 1:** Baseline characteristics and oncological data of included patients as well as results of PET/CT scan

Patient-ID	1	2	3	4	5
Age at initial diagnosis	57	60	53	54	58
Primary tumor site	Retroperitoneum	Testis	Testis	Testis	Testis
Initial therapy	Chemotherapy	Ablatio testis	Ablatio testis	Ablatio testis	Chemotherapy
Primary histopathology	Seminoma	Seminoma	NSGCT (embryonal carcinoma)	NSGCT (embryonal carcinoma)	NSGCT (teratoma, embryonal carcinoma)
Clinical stage according to UICC	IIC	IS	IIIB	IIIB	IIIB
Prognostic group according to IGCCCG	Good prognosis	Good Prognosis	Intermediate prognosis	Poor Prognosis	Intermediate Prognosis
Chemotherapy regime (cycles)	Etoposide, Cisplatin (4)	None	Ifosfamide, Etoposide, Cisplatin (4)	Ifosfamide, Etoposide, Cisplatin (4)	Ifosfamide, Etoposide, Cisplatin (4)
Suspicious tumor localization and dimensions (SAD x LAD, axial PET/CT)	A, retroperitoneal (5.2 × 5.4 cm) B, retroperitoneal (1.6 × 2.3 cm)	None	retroperitoneal (1.8 × 2 cm)	A, retroperitoneal (2.5 × 1.1 cm) B, retroperitoneal (no adequate measurement possible)	retroperitoneal (10 × 8 cm)
Histopathology of RTR-specimen	-	-	Necrosis, fibrosis, inflammation	A, necrosis B, necrosis	Teratoma
Histological FAP expression score (Specimen)	11 (needle biopsy)	32 (testis)	34 (retroperitoneal tumor mass)	0 (retroperitoneal tumor mass)	23 (retroperitoneal tumor mass)
SUV_max_	A, 4.89 (retroperitoneal tumor mass) B, 1.55 (retroperitoneal tumor mass)	No signal	6.98 (retroperitoneal tumor mass)	A, 2.72 (retroperitoneal tumor mass) B, 2.34 (retroperitoneal tumor mass)	4.22 (retroperitoneal tumor mass)
SUV_max_ after chemotherapy (SAD x LAD, axial in PET/CT)	A, 5.41 (CAVE: superimposed activity of the Vena cava inferior; 1.6 × 2.3 cm) B, 1.76 (CAVE: superimposed activity of the Vena cava inferior; 0.8 × 1 cm)	-	-	-	-

RTR = residual tumor resection; SUV_max_ = standardized uptake value; SAD = short-axis diameter; LAD = long-axis diameter

Five Patients with median age of 57 years (Interquartile Range (IQR) 54.5–59.5) were included between May 2024 and March 2025 in this pilot study. Two (40%) patients were planned for chemotherapy, three (60%) patients were planned for RTR. One patient (patient 1) who received chemotherapy was diagnosed with primary retroperitoneal GCT (prGCT). Patients received a median activity of 214 MBq (IQR 204–258). A Pearson correlation analysis revealed a positive correlation between FAP expression score and SUV_max_ (*r* = 0.80).

## Staging prior to chemotherapy

Two patients were planned for chemotherapy (patients 1 and 2). Patient 2 was diagnosed with testicular seminoma and received PET/CT scan due to marker persistence of alfa fetoprotein (AFP) after ablatio testis. Due to the stable persistence of AFP levels and unremarkable imaging findings, the patient underwent active surveillance. Likewise, no suspicious metastatic lesions were detected in conventional CT as well as with PET and therefore no SUV_max_ was calculated. Histopathology of the primary cancer showed a high FAP expression score of 34 (Table 1).

**Fig. 1:**
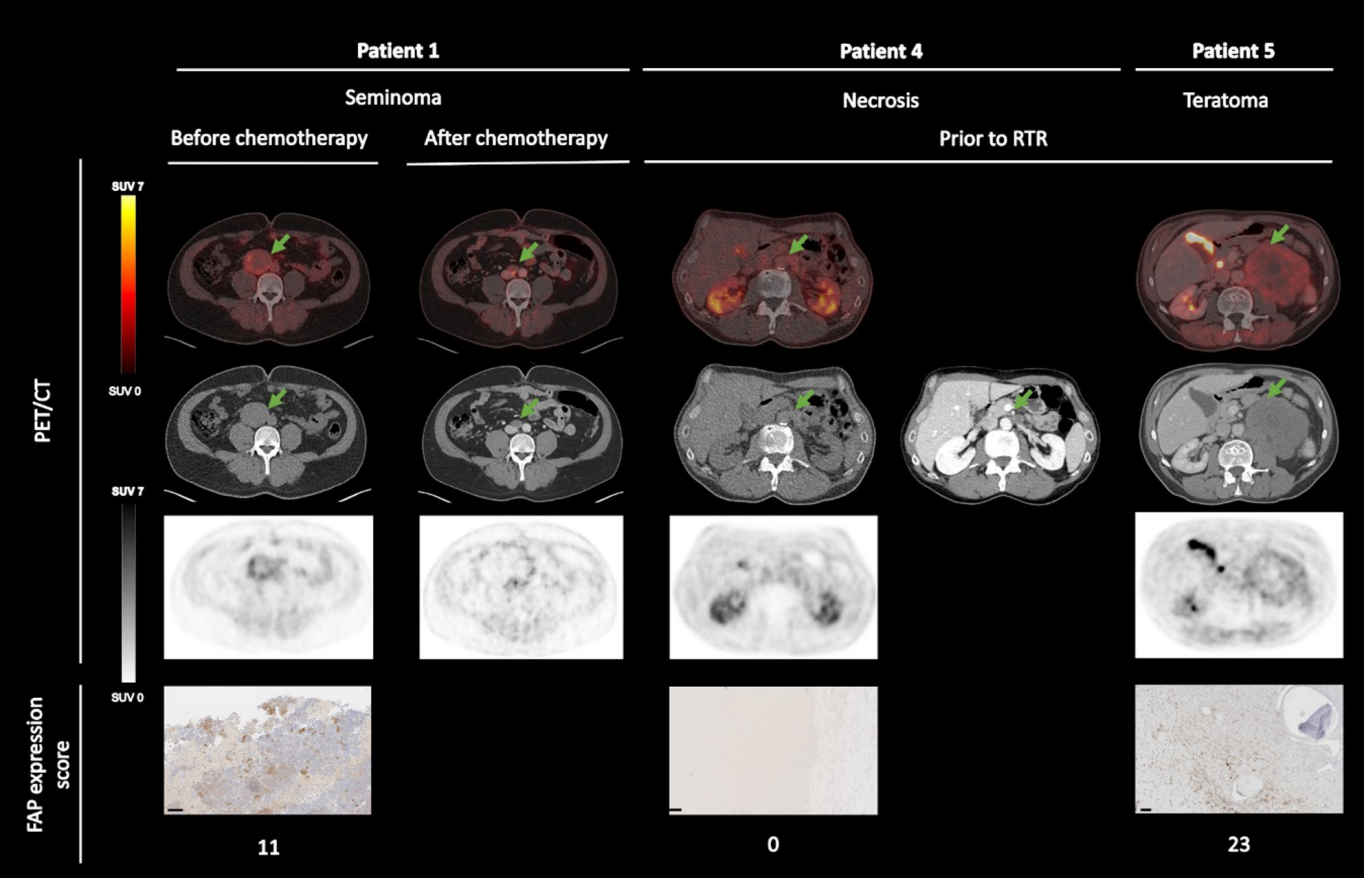
Results from PET/CT scan correlated with FAP expression score in pathology (RTR = residual tumor resection)

Patient 1 was diagnosed with seminomatous prGCT by CT-interventional biopsy and received PET/CT-scan prior and after chemotherapy with four cycles etoposide and cisplatin. Prior tumor masses measured 5.2 × 5.4 cm and 1.1 × 1.4, respectively, and showed a SUV_max_ of 4.89 and 1.55 prior to chemotherapy with 4 cycles of Etoposide and Cisplatin. Lactate dehydrogenase (LDH) was the only tumor marker elevated prior to chemotherapy with a value of 363 U/l. Histopathology of the biopsy revealed a FAP expression score of 11. The second PET/CT-scan was performed six weeks after the last of four chemotherapy cycles, showing an axial reduction of tumor mass 1.6 × 2.3 cm respectively 0.8 × 1.0 cm, a normalized LDH and a SUV_max_ of 5.41 and 1.76 (Fig. 1; Table 1), respectively. It is important to note that both lesions were near the vena cava inferior, resulting in superimposing tracer activity.

## Evaluation of residual tumor mass prior to RTR

Three patients diagnosed with testicular NSGCT who were planned for RTR after chemotherapy were included (patients 3, 4 and 5). The mean SUV_max_ was 4.07 (range 2,34–6.98). Patient 5, who was diagnosed with teratoma in the final pathology (FAP expression score 23) after RTR. Although patient 3 was diagnosed with necrosis in pathology, the scan demonstrated a SUV_max_ of 6.98 and showed a FAP expression score of 34 (Table 1). Patient 4 was diagnosed with necrosis in pathology, the scan demonstrated a SUV_max_ of 2.72 and 2.34 and showed a FAP expression score of 0 (Table 1).

## Discussion

In general, the treatment of germ cell tumors (GCT) is associated with an excellent prognosis. However, therapeutic interventions for advanced disease can result in long-term complications. Accurate tumor monitoring is crucial to balance invasive treatments with surveillance strategies. Current morphological imaging modalities and serum tumor markers have limitations in reflecting true tumor activity. [^18^F]F-FAPI-74 PET/CT represents a novel approach to GCT imaging. In this pilot study, we demonstrate that [^18^F]F-FAPI-74 PET/CT imaging of GCT across different disease stages is feasible and correlated with fibroblast activation protein (FAP) expression on histopathology (*r* = 0.8).

According to current guidelines, [^18^F]FDG PET/CT can be performed to evaluate the presence of vital residual tumor masses before RTR in seminomatous GCT, but there is no recommendation for its use in NSGCT due to low sensitivity [4, 12]. Studies show a high negative predictive value (NPV) of 94%, but also a high rate of false-positive findings in seminomatous GCT [13]. In our study, we found that one patient with poor signal on [^18^F]F-FAPI-74 PET/CT prior to RTR did not have a vital tumor mass in pathology. In addition, we included a patient diagnosed with seminomatous GCT and persistent tumor marker AFP (cS IS), who showed no evidence of tracer activity in the retroperitoneum, although measurement of FAP expression in the primary tumor specimen showed a high FAP expression score of 32. Since the patient remained without evidence of disease during follow-up and due to the high FAP expression in the primary tumor tissue, we hypothesized that metastases would also have shown higher FAP expression and therefore would have been detected by FAPI PET/CT. Regarding the atypical AFP elevation in a seminoma patient, it is important to note that non-specific AFP elevation occurs in approximately 2% of seminoma patients [14]. In line with these results, studies showed a high NPV of over 90% across other tumor entities [7]. Thus [^18^F]FAPI PET/CT might have a potential in the detection of healthy patients before RTR [7].

The results of our study also show conflicting results. One Patient undergoing post-chemotherapy RTR showed tracer uptake on PET/CT and a high FAP expression in the pathology specimen, but no signs of residual vital tumor. False-positive results go in line with a publication by Ludwig et al. suggesting that such findings might be explained by activated fibroblasts expressing FAP due to remodeling processes or fibroinflammatory conditions [15]. Kessler et al. and Taralli et al. demonstrated that false-positive results in FAPI PET/CT can be caused by healing and inflammatory processes resulting from chemotherapy [16, 17]. Next, we present a patient with prGCT showing a higher value of SUV_max_ after chemotherapy, although successful response to chemotherapy was confirmed due to decreasing retroperitoneal tumor masses and decreasing tumor markers. This might be false-positive due to overlapping activity of the vena cava inferior. Hotta et al. showed that FAPI tracer activity in blood vessels might influence the SUV_max_ of adjacent structures [18]. Another study reported that cisplatin and etoposide can induce inflammation within the tumor microenvironment [19], resulting in stronger tracer uptake after chemotherapy. Interestingly, needle biopsy of the retroperitoneal mass with higher FAP-expression showed a FAP expression score of 11 by histopathology, which is regarded as negative. However, studies showed that the expression analysis of the pathological specimen may differ between the needle biopsy and the whole tumor [20].

Teratoma inherits a special role in the treatment of advanced GCT. Teratoma is chemotherapy-resistant and does not express tumor markers [21]. Moreover, [^18^F]FDG PET/CT has proven unreliable for NSGCT overall, not just teratoma. It underperforms compared to CT when distinguishing necrosis or fibrosis from teratoma, can give false‑positive signals in post‑chemotherapy inflammation, and may miss small lesions altogether [22, 23]. Recent diagnostic approaches to predict malignancy in residual tumor masses by microRNA-371 showed promising results in identifying viable cancer in residual post-chemotherapy tumor masses but also failed in the detection of teratoma [24]. In our study, we observed a high tracer uptake at PET/CT of a residual tumor containing vital teratoma.

We acknowledge several limitations of our analysis. First, the small sample size limits the generalizability of our findings. Second, the retrospective design precluded a standardized protocol for scan timing both before surgery and after chemotherapy. Third, we employed [^18^F]F-FAPI-74 as the tracer, whereas other studies in urologic tumors—such as penile or bladder cancer—have used [^68^Ga]Ga-FAPI-46, which complicates direct comparisons [7, 8]. Prospective studies with larger cohorts are needed to determine the true strengths and weaknesses of [¹⁸F]F-FAPI-74 PET/CT in germ cell tumor patients. Furthermore, a power analysis was not performed in advance. The absence of a direct, head-to-head comparison with [^18^F]FDG PET/CT is a significant limitation.

## Conclusion

[^18^F]F-FAPI-74 PET/CT appears to be a feasible imaging method in GCT and offers molecular insights that complement conventional CT and [^18^F]FDG PET/CT. Tracer uptake frequently corresponded to fibroblast activation, such as in cases of teratoma; however, some inconsistencies were observed, including uptake in nonviable tissue and one missed marker-positive lesion. Due to the limited number of cases in this pilot study, further research is necessary to clarify the diagnostic value of FAPI PET/CT in comparison to established imaging techniques for staging and managing GCT.

## Supplementary Information

Supplementary Material 1

## Data Availability

The datasets generated during and/or analysed during the current study are available from the corresponding author on reasonable request.

## Abbreviations

AFP: Alpha fetoprotein
CT: Computed tomography
ceCT: Contrast enhanced computed tomography
FAP: Fibroblast Activation Protein
GCT: Germ cell tumor
HCG: Human chorionic gonadotropin
LAD: long-axis diameter
LDH: Lactate dehydrogenase
NSGCT: Nonseminomatous germ cell tumor
PET: Positron Emission Tomography
prGCT: Primary retroperitoneal germ cell tumor
RTR: Residua tumor resection
SAD: short-axis diameter
SUV: Standardized uptake value
TC: Testicular cancer

## References

[1] Siegel RL, Kratzer TB, Giaquinto AN, Sung H, Jemal A. Cancer statistics, 2025. CA Cancer J Clin. 2025;75:10–45. 39817679 10.3322/caac.21871 PMC11745215

[2] Singla N, Bagrodia A, Baraban E, Fankhauser CD, Ged YMA. Testicular germ cell tumors: a review. JAMA. 2025;333:793–803. 39899286 10.1001/jama.2024.27122

[3] Leitlinienprogramm Onkologie. accessed February 12, : S3-Leitlinie Diagnostik, Therapie und Nachsorge der Keimzelltumoren des Hodens: Langversion 1.1., 2020. AWMF-Registrierungsnummer: 043/049OL, 2020. https://www.leitlinienprogramm-onkologie.de/leitlinien/hodentumoren (2024).

[4] EAU. EAU Guidelines: Edn. presented at the EAU Annual congress Madrid 2025, 2025.

[5] Kunst N, Long JB, Westvold S, Sprenkle PC, Kim IY, Saperstein L, et al. Long-term outcomes of prostate-specific membrane antigen-PET imaging of recurrent prostate cancer. JAMA Netw Open. 2024;7:e2440591. 39441595 10.1001/jamanetworkopen.2024.40591 PMC11581571

[6] Nicu A-T, Medar C, Chifiriuc MC, Gradisteanu Pircalabioru G, Burlibasa L. Epigenetics and testicular cancer: bridging the gap between fundamental biology and patient care. Front Cell Dev Biol. 2022;10:861995. 35465311 10.3389/fcell.2022.861995 PMC9023878

[7] Unterrainer LM, Eismann L, Lindner S, Gildehaus F-J, Toms J, Casuscelli J, Holzgreve A, Kunte SC, Cyran CC, Menold P, Karl A, Unterrainer M, Ledderose ST, Stief CG, Bartenstein P, Kretschmer A, Schulz GB. 68 GaGa-FAPI-46 PET/CT for locoregional lymph node staging in urothelial carcinoma of the bladder prior to cystectomy: initial experiences from a pilot analysis. Eur J Nucl Med Mol Imaging. 2024;51:1786–9. 38236427 10.1007/s00259-024-06595-z PMC11043110

[8] Eismann L, Ledderose ST, Enzinger B, Berg E, Westhofen T, Rodler S, Schulz GB, Toms J, Holzgreve A, Gildehaus FJ, Brendel M, Cyran CC, Unterrainer M, Stief CG, Bartenstein P, Schlenker B, Unterrainer LM. 68GaGa-FAPI-46 PET/CT for penile cancer - a feasibility study. Eur J Nucl Med Mol Imaging. 2024;51:3461–4. 38761187 10.1007/s00259-024-06763-1

[9] Hope TA, Calais J, Goenka AH, Haberkorn U, Konijnenberg M, McConathy J, et al. SNMMI procedure standard/EANM practice guideline for fibroblast activation protein (FAP) PET. J Nucl Med. 2025;66:26–33. 39572227 10.2967/jnumed.124.269002 PMC11705787

[10] Kaplan İ, Can C, Güzel Y, Alabalik U, Kömek H. 68 GA-FAPI-04 PET/CT versus 18 F-FDG PET/CT in imaging of malignant mixed germ cell testicular tumor. Clin Nucl Med. 2023;48:e195–7. 36728220 10.1097/RLU.0000000000004530

[11] Jorgenson LC, Torbenson MS, Halfdanarson TR, Kankeu Fonkoua LA, Tran NH, Roberts LR, et al. Immunohistochemical basis for FAP as a candidate theranostic target across a broad range of cholangiocarcinoma subtypes. Front Nucl Med. 2024;4:1480471. 39664608 10.3389/fnume.2024.1480471 PMC11631625

[12] Wit Mde, Brenner W, Hartmann M, Kotzerke J, Hellwig D, Lehmann J, et al. [^18^F]-FDG–PET in clinical stage I/II non-seminomatous germ cell tumours: results of the German multicentre trial. Ann Oncol. 2008;19:1619–23. 18453520 10.1093/annonc/mdn170

[13] Treglia G, Sadeghi R, Annunziata S, Caldarella C, Bertagna F, Giovanella L. Diagnostic performance of fluorine-18-fluorodeoxyglucose positron emission tomography in the postchemotherapy management of patients with seminoma: systematic review and meta-analysis. Biomed Res Int. 2014;2014:852681. 24963486 10.1155/2014/852681 PMC4052095

[14] Dieckmann K-P, Anheuser P, Simonsen H, Höflmayer D. Pure testicular seminoma with non-pathologic elevation of alpha fetoprotein: a case series. Urol Int. 2017;99:353–7. 28668957 10.1159/000478706

[15] Ludwig V, Maliha PG, Shen J, Tonnelet D, Raman S, Litwin MS, et al. ^68^GaGa-FAPI-46 false-positive uptake after chemotherapy in nonseminomatous germ cell tumor metastatic lesions. J Nucl Med. 2024;65:1328–9. 38664018 10.2967/jnumed.124.267609

[16] Taralli S, Lorusso M, Perrone E, Perotti G, Zagaria L, Calcagni ML. PET/CT with fibroblast activation protein inhibitors in breast cancer: diagnostic and theranostic application-a literature review. Cancers (Basel). 2023. 10.3390/cancers15030908. 36765866 10.3390/cancers15030908 PMC9913570

[17] Kessler L, Ferdinandus J, Hirmas N, Zarrad F, Nader M, Kersting D, et al. Pitfalls and common findings in ^68^Ga-FAPI PET: a pictorial analysis. J Nucl Med. 2022;63:890–6. 34620730 10.2967/jnumed.121.262808 PMC9157730

[18] Hotta M, Rieger AC, Jafarvand MG, Menon N, Farolfi A, Benz MR, et al. Non-oncologic incidental uptake on FAPI PET/CT imaging. Br J Radiol. 2023;96:20220463. 35776566 10.1259/bjr.20220463 PMC9975522

[19] Behranvand N, Nasri F, Zolfaghari Emameh R, Khani P, Hosseini A, Garssen J, Falak R. Chemotherapy: a double-edged sword in cancer treatment. Cancer Immunol Immunother. 2022;71:507–26. 34355266 10.1007/s00262-021-03013-3 PMC10992618

[20] Ough M, Velasco J, Hieken TJ. A comparative analysis of core needle biopsy and final excision for breast cancer: histology and marker expression. Am J Surg. 2011;201:692–4. 20850706 10.1016/j.amjsurg.2010.02.015

[21] Farci F, Shamsudeen S. StatPearls: testicular teratoma. Treasure Island (FL). 2025. 33620805

[22] Kollmannsberger C, Oechsle K, Dohmen BM, Pfannenberg A, Bares R, Claussen CD, et al. Prospective comparison of ^18^Ffluorodeoxyglucose positron emission tomography with conventional assessment by computed tomography scans and serum tumor markers for the evaluation of residual masses in patients with nonseminomatous germ cell carcinoma. Cancer. 2002;94:2353–62. 12015760 10.1002/cncr.10494

[23] Ayati N, Askari E, Fotouhi M, Soltanabadi M, Aghaee A, Roustaei H, Scott AM. Nuclear medicine imaging in non-seminomatous germ cell tumors: lessons learned from the past failures. Cancer Imaging. 2024;24:156. 39558421 10.1186/s40644-024-00794-5 PMC11571929

[24] Dieckmann K-P, Grobelny F, Soave A, Che Y, Nestler T, Matthies C, Heinzelbecker J, Winter A, Heidenreich A, Niemzok T, Dumlupinar C, Angerer M, Wülfing C, Paffenholz P, Belge G. Serum levels of MicroRNA-371a-3p for predicting the histology of postchemotherapy residual masses of germ cell tumours. Eur Urol Focus. 2024;10:851–7. 38729824 10.1016/j.euf.2024.05.002

