# Supplementary material for: [18F]F-FAPI-74 PET/CT for gonadal and extragonadal germ cell Tumors: a pilot study
Source: EJNMMI Res. 2025 Dec 20;16:13. doi: 10.1186/s13550-025-01347-y (PMC12834867; doi:10.1186/s13550-025-01347-y)
Supplement: Supplementary file 1 — Supplementary Material 1 [file 13550_2025_1347_MOESM1_ESM.docx]

| Klinikum der Universität München · Urologische Klinik und Poliklinik Marchioninistr. 15, 81377 München |  |  |  | **Direktor**  Prof. Dr. med. Christian Stief  **Ihr Ansprechpartner**  PD Dr. med. Yannic Volz  Tel. +49 89 4400-711357  Fax +49 89 4400-75444  yannic.volz@med.uni-muenchen.de  www.lmu-klinikum.de  04.11.2025 |
| --- | --- | --- | --- | --- |
| European Journal of Nuclear Medicine and Molecular Imaging - Research |  |  |  |  |
|  | |  |  |  |

Dear Editors-in-Chief,

On behalf of all authors, we would once more like to sicerely thank you and the reviewers for the thorough evaluation of our manuscript entitled "[^18^F]F-FAPI-74 PET/CT for Gonadal and Extragonadal Germ Cell Tumors – a Pilot Study". We are grateful for the constructive feedback, which has helped us to improve the clarity, structure, and overall quality of our work.

In response to the reviewers’ comments, we carefully revised the manuscript, addressing all concerns point by point. These revisions include corrected typos and improved table 1 and figure 1.

We would like to express our appreciation for the time and effort invested by both the reviewers and the editorial team in providing such valuable feedback. We hope that our revised version now meets the high standards of *EJNMMI Research*, and we are confident that our study adds meaningful insight into a potential new imaging technology in patients suffering from germ cell tumors.

We look forward to your favorable consideration and would be happy to address any further questions or clarifications that may arise.

Thank you very much for your time and support.

Yours sincerely,

Dr. med. Marc Kidess and PD Dr. med. Michael Chaloupka

***Editor:***

**We would like to express our gratitude to the editor who considered our manuscript for publication in EJNMMI Research, as well as for taking the time to help us improve our work.**

- *Understandably, this is a very small patient cohort. I would kindly ask the authors to reformat as a short communication and further take into account the below comments from the reviewers upon submission of a revised version of the manuscript.*

**Thank you for taking the time to review our manuscript. We have resubmitted it as a short communication.**

***Reviewer 1:***

**We would like to begin by sincerely thanking Reviewer 1 for the thorough evaluation of our manuscript and for providing valuable comments and suggestions that have helped us improve its overall quality.**

- SUVmax
  Please adjust accordingly --> SUVmax subscript

**Thank you very much for helping us to improve our manuscript. We changed the spelling according to your recommendation.**

- Background p.6
  16f. PET imaging has the advantage of combining conventional imaging with molecular imaging to detect occult metastases in other urologic malignancies. [5].
  --> … other urologic malignancies [5].

**Thank you very much for your remark, we corrected our mistake.**

- 22f. FAP can be targeted by several tracers and the use of FAPI PET/CT has shown promising results for staging in other malignancies like bladder cancer [7] or penile cancer [8].
  --> … like bladder cancer or penile cancer [7,8].

**Thank you very much for your remark. We have changed this accordingly.**

- Discussion p.8
  56f vital residual tumor masses before RTR in seminomatous GCT[4], but there is no recommendation for its use in NSGCT due to low sensitivity [12].
  -->.… GCT, … [4,12].

**Thank you for this remark. We have changed this accordingly.**

- p.9
  8f. Thus [18F]FAPI PET/CT might have a potential in the detection of healthy patients before RTR. [7].
  -->.… RTR [7].

**Thank you very much for your remark. We corrected our mistake accordingly.**

- 35ff. It underperforms compared to CT when distinguishing necrosis or fibrosis from teratoma, can give false‐positive signals in post‐chemotherapy inflammation, and may miss small lesions altogether. [22, 23].
  --> … small lesions altogether [22, 23].

**We changed the wording according to your recommendation.**

- Figure Captions:
  Figure1: Results from PET/CT scan correlated with FAP expression score in pathology.
  --> Figure 1:…

**Thank you very much for your remark. We corrected our mistake accordingly.**

- Table 1
  Please do not use all capital letters

**Thank you very much for raising this point, we changed the spelling according to your recommendation.**

- Table 1: Baseline characteristics and oncological Data of included patients as well as results of PET/CT scan (RTR = Residual tumor resection; SUVmax = Standardized uptake value; SAD = short-axis diameter; LAD = long-axis diameter.
  --> Table 1: Baseline characteristics and oncological data…

**Thank you for this remark. We have changed this accordingly.**

- (RTR = Residual tumor resection; SUVmax = Standardized uptake value; SAD = short-axis diameter; LAD = long-axis diameter.
  --> (RTR = residual tumor resection; SUVmax = standardized uptake value; SAD = short-axis diameter; LAD = long-axis diameter.)

**Thank you very much for raising this point, we changed the spelling according to your recommendation.**

- PRIMARY THERAPY
  Sounds somewhat unusual ◊Initial therapy/First line therapy??

**Thank you for raising this important point. To make this clearer, we changed the wording to “Initial therapy”.**

- PROGNOSIS GROUP ACCORDING TO IGCCCG
  --> PROGNOSTIC…

**Thank you very much for raising this point, we changed the spelling according to your recommendation.**

- (CYCLES 4)
  --> (4)
  Once defined, it should not be repeated thereafter

**Thank you very much for raising this point, we changed the spelling according to your recommendation.**

- LOCALIZATION SUSPICIOUS TUMOR MASS (SAD AXIAL X LAD AXIAL) IN PET/CT
  --> Suspicious tumor localization and dimensions (SAD × LAD, axial PET/CT)

**Thank you very much for raising this point, we changed the spelling according to your recommendation.**

- SUVMAX SUSPICIOUS TUMOR MASS
  --> SUVmax (Please delete the remaining text)

**We changed the wording according to your recommendation.**

- SUVMAX SUSPICIOUS AFTER CHEMOTHERAPY (SAD AXIAL X LAD AXIAL)
  --> SUVmax after … (SAD X LAD, axial)

**We changed the wording according to your recommendation.**

- Figure 1
  RTR
  --> Please define in the figure legend

**Thank you very much for giving us the opportunity to improve the quality of our manuscript, we defined RTR in the figure legend.**

- please also include a grayscale scale bar for the PET signal

**Thank you for mentioning this very important point. We have now included a grayscale bar in figure 1.**
